# Short-Term Outcomes of Ethnic and Racial Minority Pediatric Patients Following Traumatic Brain Injury in the State of Texas

**DOI:** 10.7759/cureus.16050

**Published:** 2021-06-30

**Authors:** Colin Son, Izabela Tarasiewicz

**Affiliations:** 1 Neurosurgery, Neurosurgical Associates of San Antonio, San Antonio, USA; 2 Neurosurgery, University of Texas Health Science Center at San Antonio, San Antonio, USA

**Keywords:** racial disparity, pediatric trauma, traumatic brain injury, pediatric head trauma

## Abstract

Background

Pediatric traumatic brain injury represents a significant cause of morbidity and mortality. Broadly healthcare disparities exist for ethnic and racial minorities in the United States but it has not previously be evaluated how these disparities might influence outcomes in pediatric traumatic brain injury.

Methods

We sought all hospital admissions between the years 2006 and 2011 for patients aged 0-17 years admitted with traumatic brain injuries as identified by the International Classification of Diseases, 9th Revision (ICD-9) code, from a statewide database of all civilian hospital admissions. Demographic information including race, ethnicity, insurance status and illness severity as calculated by All Patient Refined-Diagnosis Related Group (APR-DRG) were analysed versus the disposition at discharge.

Results

14,087 pediatric traumatic brain injury patients were admitted between 2006 and 2011. Pediatric traumatic brain injury patients of ethnic or racial minority had higher rates of in-hospital mortality as compared to whites (4.2% versus 3.3%, p = 0.009) and were less likely to be discharged to inpatient rehabilitation (2.9% versus 4%, p < 0.001). These disparities persisted even when controlling for insurance status and illness severity.

Conclusion

Ethnic and racial minority children from the U.S. state of Texas suffer worse short-term outcomes following traumatic brain injury than their white counterparts. Strategies are needed for addressing this disparity.

## Introduction

Disparities in outcomes for the United States ethnic and racial minorities have been demonstrated in a host of medical conditions including traumatic brain injury. Work has been done on the short- and long-term outcomes and care received for adult ethnic and racial minorities suffering from traumatic brain injuries. Adult African-Americans have significantly higher in-hospital mortality and poorer long-term outcomes than their white counterparts [1]. Adult minorities in the U.S. are less likely to return to work following traumatic brain injuries than whites [2]. Adult minorities in the U.S. suffering mild traumatic brain injuries may receive disparate emergency department care as compared to whites [3]. Despite such work, less has been done to the look at the influence of ethnicity or race on outcomes following traumatic brain injury in pediatric populations.

In the United States, children with traumatic brain injuries account for more than five hundred thousand emergency department visits per year, fifty thousand hospital admissions per year and more than $2 billion in health care charges [4-6]. Trauma remains a leading cause of mortality for patients younger than eighteen years of age in the United States [7]. And, amongst pediatric trauma victims, traumatic brain injury is the most common cause of death [8]. Traumatic brain injury in pediatric patients, even mild traumatic brain injury, results in significant long-term morbidity [9-12]. 

The work that has been done on race and ethnicity in pediatric traumatic brain injury shows that some ethnic and racial minority groups have higher incidences of pediatric traumatic brain injury than whites [4]. They may be less likely to be admitted to the hospital following traumatic brain injury [13]. African-American children may have an increased rate of in hospital mortality following traumatic brain injury as compared to whites [4]. Still, the role of racial and ethnic minority status in outcomes following pediatric traumatic brain injury remains understudied. To our knowledge, no study has assessed outcomes of non-white, non-African-American minority children following traumatic brain injury in a categorical way. And no study since the work of Langlois, et al has looked again at the outcomes for all minority victims of pediatric traumatic brain injuries, including African-Americans [4].

Our work here presents the incidence and short-term outcomes for pediatric traumatic brain injury victims by ethnicity and race over a five-year period, from 2006 to 2011, in the United States’ state of Texas.

## Materials and methods

The Texas Inpatient Public Use Data File is the second-largest state-level data set of hospital discharges in the United States [14]. It contains a record of every discharge from civilian hospitals within the state of Texas. Amongst the database are a number of data points to include, pertinent to this study: age, race, ethnicity, sex, disposition at discharge, diagnoses by International Classification of Diseases, 9th Revision (ICD-9) code, length of stay, total charges, location presenting from, Diagnosis-Related Group (DRG) code, illness severity as calculated by All Patient Refined-Diagnosis Related Group (APR-DRG) codes and insurance status [15-17].

The database was queried for all hospital discharges related to an admission diagnosis of traumatic brain injury by ICD-9 code between 2006 and 2011. The ICD-9 codes queried appear in Table 1.

**Table 1:** International Classification of Diseases, Ninth Revision traumatic brain injury codes.

ICD-9 codes	Description
800.0 – 801.3	Fracture of the vault or base of the skull
803.0 – 804.3	Other and unqualified multiple fractures of the skull
850 – 854.1	Intracranial injury, including concussion, contusion, laceration and hemorrhage
873.0 – 873.9	Other open wound to the head

This initial query was then weaned to include only those patients less than 18 years of age and further reduced to include only discharges from acute care hospitals.

Patients were classified by race and ethnicity, the latter a bimodal variable identifying patients as Hispanic or non-Hispanic. Those identified as Hispanic, no matter their identified race, were included as such in the analysis and discarded from the race category. The combined classification of race and ethnicity, as defined in the Texas Inpatient Public Use Data File, thus included: Native American, Asian, black, Hispanic, non-Hispanic white, other. Because the Texas Inpatient Public Use Data File contains reported data from more than a thousand hospitals, the methods of identification of race and ethnicity varied from reporting site to reporting site. Depending on the reporting hospital the identification of ethnicity and race was based on self classification or observation by those collecting the data.

2010 U.S. census data was used to identify population sizes of those under 18 years of age for various ethnic and racial groups in Texas [18]. This data was used to calculate annual incidence rates.

Analysis was carried out on Stata software (StataCorp LP, College Station, TX). Pearson Chi-squared tests were used to assess statistical significance. Comparisons of the disposition at discharge, insurance status, total charges and illness severity were made amongst the various combined ethnic and racial groups and amongst all minorities and non-Hispanic whites. Logistic regression was carried out to control for insurance status, insurance type and illness severity.

## Results

Between 2006 and 2011, 97,931 patients were discharged from Texas civilian acute care hospitals with a traumatic brain injury. Of these patients, 14,087 were less than eighteen years of age. The demographics of pediatric traumatic brain injury in the US state of Texas appear in Table 2.

**Table 2:** Demographics of pediatric traumatic brain injury in Texas, 2006-2011. APR-DRG: All Patient Refined-Diagnosis Related Group; CHIP: Children's Health Insurance Program.

Characteristics	Number of patients
Age	
1-28 days	178 (1.3%)
29-365 days	2593 (18.4%)
1-4 years	3068 (21.8%)
5-9 years	2177 (15.5%)
10-14 years	2690 (19.1%)
14-17 years	3381 (24%)
Sex	
Male	9039 (64.1%)
Female	5045 (35.8%)
Race or Ethnicity	
Native American	208 (1.5%)
Asian	187 (1.3%)
Black	1457 (10.3%)
Hispanic	5476 (38.8%)
Non-Hispanic White	6017 (42.7%)
Other	742 (5.3%)
APR-DRG Illness Severity	
1	6367(45.2%)
2	4082 (29%)
3	2099 (14.9%)
4	1539 (10.9%)
5	
Uninsured	1126 (7.9%)
Medicaid/CHIP	6136 (43.6%)
Medicare	32 (0.2%)
Liability	531 (3.8%)
TRICARE/Other Federal	428 (3%)
Private	5654 (40.1%)
Other	180 (1.3%)

Using 2010 census data for the US state of Texas the per annum rate of acute hospitalization for pediatric traumatic brain injury was 28.1 per 100,000. By race and ethnicity, the rates were 25.6 per 100,000 for Hispanics, 24.4 per 100,000 for blacks, 13.8 per 100,000 for Asians, 26.1 per 100,000 for all minorities versus 32.1 per 100,000 for non-Hispanic whites. The variations in the annual rate of acute hospitalization for traumatic brain injury was significant amongst all minorities versus non-Hispanic whites (p < 0.001). An annual rate was unable to be calculated for those of Native American race.

Of the 14,087 discharges, 545 children expired during their hospital stay for a rate of 3.8%. The demographics of in-hospital mortality can be found in Table 3.

**Table 3:** In-hospital mortality following pediatric traumatic brain injury in Texas, 2006-2011. APR-DRG: All Patient Refined-Diagnosis Related Group; CHIP: Children's Health Insurance Program.

Characteristics	Expired	Survived
Age		
1-28 days	5 (2.8%)	173
29-365 days	72 (2.8%)	2521
1-4 years	134 (4.4%)	2934
5-9 years	55 (2.5%)	2122
10-14 years	101 (3.8%)	2589
14-17 years	178 (5.3%)	3203
Sex		
Male	351 (3.9%)	8688
Female	193 (3.8%)	4852
Race or ethnicity		
Native American	8 (3.8%)	200
Asian	2 (1.1%)	185
Black	73 (5%)	1384
Hispanic	200 (3.7%)	5276
Non-Hispanic White	203 (3.3%)	5814
Other	59 (8%)	683
APR-DRG Illness Severity		
1	5 (0.08%)	6362
2	26 (0.64%)	4056
3	158 (7.5%)	1941
4	356 (23.1%)	1183
Insurance		
Uninsured	66 (5.9%)	1060
Medicaid/CHIP	250 (4.1%)	5886
Medicare	1 (3.1%)	31
Liability	22 (4.1%)	509
TRICARE/Other Federal	16 (3.7%)	412
Private	174 (3.1%)	5480
Other	16 (8.9%)	164

Non-Hispanic white patients had a significantly lower rate of in-hospital mortality than blacks (p = 0.003) and minorities as a whole (p = 0.009) but not compared to patients of Native American (p = 0.711) or Hispanic (p = 0.418) heritage. Children categorized as Asian had non-significant lower in-hospital mortality as compared to other ethnicities and races; versus non-Hispanic whites (p = 0.083). In addition to the differences in in-hospital mortality, those minority patients who survived were significantly less likely to receive referral to inpatient rehabilitation on discharge (4% versus 2.9%, p < 0.001). The significance of minority status on mortality and discharge to inpatient rehabilitation persisted even when using logistic regression to control for insured status, insurance type and illness severity.

The difference in rates of insurance for non-Hispanic whites versus ethnic and racial minorities (93.1% versus 91.2%) was not significant (p = 0.061) but minority patients were significantly more likely to be covered by Medicaid or as part of the Children’s Health Insurance Program (CHIP) than their non-Hispanic white counterparts (54.4% versus 29%, p < 0.001) and less likely to be covered by private insurance (29% versus 55%, p < 0.001).

The illness severity and total charges were similar amongst all racial and ethnic groups.

## Discussion

Our findings show that, in the U.S. state of Texas, ethnic and racial minority pediatric patients have higher rates of in-hospital mortality following traumatic brain injury than non-Hispanic white pediatric patients. Those minority pediatric patients who survive are more likely to be discharged directly to home and less likely to receive the benefit of inpatient rehabilitation. These differences persist even when accounting for illness severity, insurance status and insurance type.

Previous work has demonstrated that African-American pediatric patients have higher rates of in-hospital mortality as compared to non-Hispanic whites [4]. To our knowledge, this is the first study to demonstrate a difference in in-hospital mortality and disposition at discharge amongst all ethnic and racial pediatric minorities versus non-Hispanic whites. In addition, it appears the first study of the epidemiology of U.S. pediatric traumatic brain injury to attempt to control for insurance status or illness severity in looking at ethnic and racial disparities. Our findings parallel the findings of other studies of disparities in U.S. adult traumatic brain injury [1,19]. 

The estimates of the incidence of pediatric traumatic brain injury vary wildly in the literature [4,20-24]. Our data includes only those cases of pediatric traumatic brain injury which resulted in hospital admission and does not count emergency room or extra-hospital presentations of pediatric traumatic brain injury. Still, our estimate of the annual rates of hospitalization even for pediatric traumatic brain injury, 28,1 per 100,000, is considerably lower compared to rates posited by others [13,24,25]. Some, perhaps much, of this difference may be explained by geographic variation, differences in the definition of diagnosis, inclusion criteria differences and double counting of admissions occurring in some datasets [26]. In addition, the continuing decline in the incidence of traumatic brain injury amongst US children may partially explain the difference [24,25]. 

The etiologies of disparities following pediatric traumatic brain injuries in ethnic and racial minorities are not entirely clear. Injury severity was similar amongst all populations. Insured status, those with any payer versus those uninsured, was similar as well. The minority populations in this study were more likely to be insured by Medicaid and less likely to have private insurance. In previous studies, insurance type has questionably been associated with variations in outcomes and discharge location following traumatic brain injuries in adults [27-29]. In our study Medicaid status was independently associated with an increased risk of in-hospital mortality versus all other payers (4.1% versus 3.3%, p = 0.023) and independently associated with a decreased rate of discharge to inpatient rehabilitation (2.9% versus 4%, p < 0.001). This partially explains disparities in our ethnic and racial minority groups. However, controlling for insurance type, along with illness severity and insurance status, still yielded significance for minority status for in-hospital mortality (p = 0.043) and discharge to inpatient rehabilitation (p = 0.002).

Disparities in health care outcomes for ethnic and racial minorities in the U.S. have been explained by differences in socioeconomic status, variations in insurance status, residential segregation, language barriers, health literacy, differences in rates of health care utilization and frank bias [30-33]. Many of these factors, beyond the scope of this particular study, may be contributing to our observed differences in the rates of in-hospital mortality and discharge to inpatient rehabilitation following pediatric traumatic brain injury.

This study represents data from a single state which may limit its generalizability. However, the Texas Inpatient Public Use Data File is the second-largest statewide all discharge database in the United States. As well, the demographics of the pediatric population in Texas includes a significant minority population. Texas’ population under the age of 18 are majority-minority [18]. The U.S. state of Texas’ demographics reflect the future of the U.S as a whole [34]. As such, the Texas Inpatient Public Use Data File is an important resource for the study of ethnic and racial variations in health care outcomes, including for the population of pediatric traumatic brain injury.

## Conclusions

Racial and ethnic minority children in the U.S. state of Texas have higher in-hospital mortality and are less likely to be discharged to inpatient rehabilitation following traumatic brain injury. The significance of minority status on these outcomes persists even when controlling for insurance status, insurance type and illness severity. More study is needed to identify the etiologies of these disparities and to develop strategies for improving the care of minority children presenting with traumatic brain injury.

## References

[1] Bowman SM, Martin DP, Sharar SR, Zimmerman FJ (2007). Racial disparities in outcomes of persons with moderate to severe traumatic brain injury. Med Care.

[2] Sherer M, Nick TG, Sander AM (2003). Race and productivity outcome after traumatic brain injury: influence of confounding factors. J Head Trauma Rehabil.

[3] Bazarian JJ, Pope C, McClung J (2003). Ethnic and racial disparities in emergency department care for mild traumatic brain injury. Acad Emerg Med.

[4] Langlois JA, Rutland-Brown W, Thomas KE (2005). The incidence of traumatic brain injury among children in the United States: differences by race. J Head Trauma Rehabil.

[5] Schneier AJ, Shields BJ, Hostetler SG, Xiang H, Smith GA (2006). Incidence of pediatric traumatic brain injury and associated hospital resource utilization in the United States. Pediatrics.

[6] Shi J, Xiang H, Wheeler K, Smith GA, Stallones L, Groner J, Wang Z (2009). Costs, mortality likelihood and outcomes of hospitalized US children with traumatic brain injuries. Brain Inj.

[7] (2015). National Center for Health Statistics. Underlying Causes of Death 1999-2014. http://wonder.cdc.gov/ucd-icd10.html.

[8] Tepas JJ, DiScala C, Ramenofsky ML (1990). Mortality and head injury: the pediatric perspective. J Pediatr Surg.

[9] Limond J, Dorris L, McMillan TM (2009). Quality of life in children with acquired brain injury: parent perspectives 1-5 years after injury. Brain Inj.

[10] Hamilton NA, Keller MS (2010). Mild traumatic brain injury in children. Semin Pediatr Surg.

[11] Rivara JB, Jaffe KM, Polissar NL (1994). Family functioning and children’s academic performance and behavior problems in the year following traumatic brain injury. Arch Phys Med Rehabil.

[12] Slomine BS, McCarthy ML, Ding R (2006). Health care utilization and needs after pediatric traumatic brain injury. Pediatrics.

[13] McCarthy ML, Serpi T, Kufera JA, Demeter LA, Paidas C (2002). Factors influencing admission among children with a traumatic brain injury. Acad Emerg Med.

[14] (2015). Texas Inpatient Public Use Data File. https://www.dshs.texas.gov/thcic/hospitals/Inpatientpudf.shtm.

[15] World Health Organization (1978). World Health Organization. International Classification of Diseases, Ninth Revision. International Classification of Diseases, Ninth Revision.

[16] (2010). Centers for Medicare and Medicaid Services. List of Diagnosis Related Groups (DRGS). FY.

[17] (2003). 3M Health Information Systems. All Patient Refined Diagnosis Related Groups, Version 20. https://www.hcup-us.ahrq.gov/db/nation/nis/APR-DRGsV20MethodologyOverviewandBibliography.pdf.

[18] (2010). United States Census 2010. https://www.census.gov/prod/cen2010/cph-2-1.pdf.

[19] Gary KW, Arango-Lasprilla JC, Stevens LF (2009). Do racial/ethnic differences exist in post-injury outcomes after TBI? A comprehensive review of the literature. Brain Inj.

[20] Kraus JF, Fife D, Cox P, Ramstein K, Conroy C (1986). Incidence, severity, and external causes of pediatric brain injury. Am J Dis Child.

[21] Rivara FP (1994). Epidemiology and prevention of pediatric traumatic brain injury. Pediatr Ann.

[22] Keenan HT, Bratton SL (2006). Epidemiology and outcomes of pediatric traumatic brain injury. Dev Neurosci.

[23] Durkin MS, Olsen S, Barlow B, Virella A, Connolly ES Jr (1998). The epidemiology of urban pediatric neurological trauma: evaluation of, and implications for, injury prevention programs. Neurosurgery.

[24] Thurman D, Guerrero J (1999). Trends in hospitalization associated with traumatic brain injury. JAMA.

[25] Asemota AO, George BP, Bowman SM, Haider AH, Schneider EB (2013). Causes and trends in traumatic brain injury for United States adolescents. J Neurotrauma.

[26] Langlois J, Rutland-Brown W, Thomas K (2004). Traumatic brain injury in the United States; emergency department visits, hospitalizations, and deaths. Center for Disease Control.

[27] Selassie AW, Pickelsimer EE, Frazier L Jr, Ferguson PL (2004). The effect of insurance status, race, and gender on ED disposition of persons with traumatic brain injury. Am J Emerg Med.

[28] Chan L, Doctor J, Temkin N, MacLehose RF, Esselman P, Bell K, Dikmen S (2001). Discharge disposition from acute care after traumatic brain injury: the effect of insurance type. Arch Phys Med Rehabil.

[29] Lehmkuhl D, Hall K, Mann N (1993). Factors that influence costs and length of stay of persons with traumatic brain injury in acute care and inpatient rehabilitation. J Head Trauma Rehabil.

[30] Fiscella K, Franks P, Doescher MP, Saver BG (2002). Disparities in health care by race, ethnicity, and language among the insured: findings from a national sample. Med Care.

[31] Williams DR, Mohammed SA (2009). Discrimination and racial disparities in health: evidence and needed research. J Behav Med.

[32] Williams DR, Collins C (2001). Racial residential segregation: a fundamental cause of racial disparities in health. Public Health Rep.

[33] Schneider EC, Zaslavsky AM, Epstein AM (2002). Racial disparities in the quality of care for enrollees in medicare managed care. JAMA.

[34] Humes K, Jones N, Ramirez R (2011). Overview of Race and Hispanic Origin. Overview of Race and Hispanic Origin.

